# De-epithelialized free gingival graft versus subepithelial connective tissue graft in the treatment of gingival recession: a systematic review and meta-analysis

**DOI:** 10.4317/medoral.27184

**Published:** 2025-08-16

**Authors:** Joyce Rebeca Ignacio Tejedo, Rebeca Alexandra Cruzado Jara, Lorena Zegarra Caceres, Andrea Vergara-Buenaventura, Francisco Wilker Mustafa Gomes Muniz, Marcelo Faveri, Jonathan Meza-Mauricio

**Affiliations:** 1 School of dentistry, Scientific University of the South, Lima, Peru; 2 Division of Periodontology, Faculty of Dentistry and Graduate School of Medical and Dental Sciences, Niigata University; 3 Department of Periodontology, Federal University of Pelotas, Pelotas, Brazil; 4 Department of Periodontology and Oral Implantology, Dental Research Division, University of Guarulhos, SP, Brazil

## Abstract

**Background:**

The best technique to harvest gingival graft to treat gingival recessions (GR) remains a topic of ongoing debate. This systematic review aimed to evaluate the effect of de-epithelialized free gingival graft (DFGG) compared to subepithelial connective tissue graft (SCTG) in the treatment of GR Miller Class I and II or Cairo type I.

**Material and Methods:**

Five databases were searched up to June 2024 to include randomized clinical trials (RCTs) comparing the clinical effects of DFGG with SCTG in the treatment of GR. The random effects model of mean differences was used to determine GR, and gain in keratinized tissue width (KTW), gingival thickness (GT) and clinical attachment level (CAL). The risk ratio was used to complete root coverage (CRC) at 6 and 12 months.

**Results:**

Five RCTs including 183 and 111 GR at 6 and 12 months were included in this systematic review. The meta-analysis showed no statistically significant difference in GR reduction, gain in KTW, GT, CRC or CAL between groups at 6 and 12 months.

**Conclusions:**

At 12 months, the clinical results of DFGG were similar to those of SCTG in the treatment of GR.

** Key words:**Gingival recession, coronal advancement flap, tunnel techniques, de-epithelialized gingival graft, subepithelial connective tissue graft.

## Introduction

Gingival recession (GR) can affect individuals with good and poor oral hygiene, as well as patients with advanced periodontal disease (1). GR is a process inducing evident loss of periodontal tissue in which the gum gradually migrates apically, exposing the root surface (2).

The etiology of GR is multifactorial and includes predisposing factors such as a thin gingival phenotype, traumatic brushing and inadequate plaque control, iatrogenic dental treatments, and parafunctional habits, among others (3,4). Clinically, as GR progresses, it can cause dental hypersensitivity, non-carious cervical lesions, and compromise the esthetics and functionality of the patients (5,6).

Currently, a high prevalence of single and multiple GRs has been reported, making their treatment a challenge for periodontists (5,7). Therefore, in the treatment of GR, it is important to consider predisposing anatomical characteristics, such as a thin gingival phenotype or limited keratinized tissue, depth of the vestibule, and high frenulum insertion (8,9).

The coronal advancement flap (CAF) and tunnel (TUN) techniques have demonstrated favorable results with respect to the root coverage procedure (10,11). CAF in association with autogenous graft is considered the gold standard (12). These grafts can be harvested with different techniques (13,14).

The de-epithelialized free gingival graft (DFGG) is obtained from the palatal area as a free gingival graft, which is de-epithelialized extraorally. This technique allows graft extraction regardless of the thickness of the palatal mucosa (15). Likewise, the connective tissue obtained through the DFGG technique has a smaller amount of adipose tissue and a greater amount of lamina propria and is therefore considered a more sTable graft (16). However, healing of the palatal wound occurs by secondary intention, which increases postoperative morbidity (17).

On the other hand, the technique of harvesting the subepithelial connective tissue graft (SCTG) is predicTable and versatile for the treatment of GR (18). Harvesting of the graft with removal of the epithelium allows the replacement of the palatal flap and primary closure (15). However, unlike DFGG, SCTG should be avoided in patients with a thin palatal area (15,17).

The DFGG has been shown to be less prone to postoperative shrinkage, because of the greater amount of connective tissue rich in collagen obtained from the lamina propria (19). On the other hand, on being tissue closer to the bone, the SCTG may contain more adipose tissue, causing less stability in the recipient area and leading the graft to contract, which is unfavorable for vascular regeneration (17).

In recent years, several randomized clinical studies have compared the use of DFGG with SCTG in the treatment of GR (8,14,17), with contradictory results. To our knowledge, only one systematic review (SR) has compared the two types of grafts in the treatment of GR. However, this SR (15) analyzed studies that compared the two types of graft independently (different studies). In that sense, the literature lacks synthesized information for direct comparison between SCTG and DFGG. Therefore, the objective of the present SR was to compare the clinical efficacy of DFGG with SCTG in the treatment of GR.

## Material and Methods

- Protocol and registration.

This SR was conducted in accordance with the Preferred Reporting Items for Systematic Reviews and Meta-Analysis (PRISMA) statement (20). The protocol for this SR was registered in the International Prospective Register of Reviews (PROSPERO) with the number CRD 42023442879.

- Focused question

In patients with GR class I and II of Miller or type I of Cairo, what are the clinical effects of the DFGG compared to the SCTG, in terms of reduction in GR, gain in keratinized tissue width (KTW), gingival thickness (GT), complete root coverage (CRC), and in clinical attachment level (CAL), and patient-related outcomes?

- Eligibility criteria

The inclusion criteria were based on the PICOS strategy (21-22). Only studies meeting the following criteria were included:

- Inclusion criteria (PICOS)

Population: Adult patients (≥18 years old) with Miller class I and II (23) or Cairo type I (24) GR who had undergone root coverage procedures. There were no restrictions in ethnicity, gender, or root coverage technique.

Intervention: Root coverage procedure with the use of DFGG.

Comparison: Root coverage procedure with the use of SCTG.

Outcome: GR reduction (primary outcome variable), gain in KTW, GT, CRC, and in CAL and patient-related outcomes (secondary outcome variables) at 6 and 12 months.

Study design: Randomized clinical trials (RCT).

- Exclusion criteria

i.Studies with insufficient information about the study design.

ii.Studies with less than 10 participants per group.

iii.Studies that included individuals with any systemic disease.

- Search strategy

The MEDLINE (PubMed), Embase, Scopus, Cochrane Library (CENTRAL), and Web of Science databases were searched in the period up to June 2024 by two independent reviewers (J.M.M. and L.A.Z.C.). The complete search strategies for the databases are presented in (Supplement 1). Furthermore, a manual search of relevant primary sources related to the topic was made in Journal of Dental Research, Journal of Clinical Periodontology, Journal of Periodontology, Journal of Periodontal Research, and Clinical Oral Investigations. Finally, the references of the studies included were explored to capture any potential additional records, as suggested by Greenhalgh and Peacock (25).

Data collection, extraction, and management

- Screening and selection of papers

Two calibrated reviewers (J.R.I.T. and R.A.C.J.), tested by the Cohen’s kappa test (26), independently screened titles and abstracts for inclusion in the databases using Rayyan Systems Inc. (https://www.rayyan.ai/). After identifying potentially relevant studies, full-text articles were obtained. Any disagreement was solved by consensus with a third reviewer (J.M.M.).

- Search outcomes and evaluation

The studies that fulfilled the eligibility criteria were processed for data extraction conducted by the two independent researchers (J.R.I.T. and R.A.C.J.), using an electronic spreadsheet (Word, Microsoft Corporation, Washington, USA). Disagreements were resolved by consensus with a third reviewer (A.V.B.). In the event of missing data, a request was sent to the authors by e-mail. For each study selected, the following variables were collected: name of author(s), year of publication, country of publication, study design, GR defect, number of patients/teeth, gender, age, intervention (number of sites in each experimental group), clinical parameters evaluated, patient reported outcomes measures (PROMS), follow-up period, and the main findings for all outcomes of interest.

- Risk of bias in individual studies

Two reviewers (J.R.I.T. and R.A.C.J.) assessed the risk of bias in the studies selected, using the Cochrane risk-of-bias tool, RoB 2 (version 2, available at: https://www.riskofbias.info/welcome/rob-2-0-tool/current-version-of-rob-2).The authors of this SR decided to assess the result related to “assignment to intervention (the intention to treat effect)” and five domains were examined: i) bias arising from the process of randomization and allocation concealment, ii) bias due to deviations from intended interventions that involved masking of participants and the team of researchers, iii) bias due to missing outcome data, iv) bias in the measurement of the outcome, and v) bias in selection of the result reported (27). Based on the responses to the signaling questions and algorithms of this tool, each domain was judged to have “low risk of bias”, “some concerns relating to the risk of bias,” or “high risk of bias”. Studies were categorized as being at low risk of bias (all domains were at low risk of bias), high risk of bias (one or more domains were at high risk of bias), some concerns (if one or more domains had some concerns) (27). Disagreements were resolved by discussion, consulting a third reviewer (M.F).

- Data synthesis and synthesis of the results

One author (F.W.M.G.M.) was responsible for statistical data collection and analysis. Ten meta-analyses were performed considering the mean difference (MD) between baseline and two different follow-ups (6 and 12 months) for each outcome (GR, KTW, GT, CAL and probing depth [PD). Two meta-analyses were performed considering the risk ratio (RR) at 6 and 12 months for CRC.

The RevMan software (version 5.3 for Mac) was used to perform both meta-analyses. Heterogeneity was assessed with the Q test and quantified by I2. As the methodological characteristics differed among the studies included, both analyses were performed using a random-effect model. Statistical significance was established as *p*<0.05.

- Certainty of the evidence

The certainty of the evidence was evaluated by the GRADE approach (28,29). This evaluation was performed for each meta-analysis. Overall, the risk of bias, inconsistency, indirectness, imprecision, and other aspects were considered to determine the certainty of the evidence. Independent analyses of both outcomes were performed (GR at 6 and 12 months of follow-up), and a summary of the findings was prepared.

## Results

- Study Selection

The electronic search strategy identified 3,928 titles. After removing duplicates, 2481 records were screened on the basis of the title and abstract. Full-text assessment was performed for 15 articles. Among these, 10 studies were excluded as they did not fulfill the eligibility criteria (Supplement 2)(30-36). Therefore, five studies were included in the present SR (Supplement 3). The reviewers showed excellent agreement (K=0.91).

- Characteristics of the studies included

The reports included were five RCTs two (37,38) with a split-mouth design and three (8,14,17) with a parallel design, which were conducted between 2010 and 2023. The main methodological characteristics of studies included are presented in Table 1. The countries of origin of each article were as follows: a clinical study in Italy (14), one in Brazil (37), one in Turkey (8), one in Egypt (17), and one in Iran (38).

Of all the clinical studies included in this SR, four studies used the CAF technique (14,17,37,38) and one study used the TUN technique (8). Data were collected from 183 and 111 Miller class I and II (Cairo type I) GRs analyzed over a period of 6 and 12 months after the intervention, respectively. Four studies (8,17,37,38) performed the analysis at 6 months. In the experimental group (DFGG), 92 teeth with GR were analyzed, and in the control group (SCTG), 91 teeth were analyzed. On the other hand, two studies (8,14) performed the analysis at 12 months in 55 and 56 teeth with GR in the test and control groups, respectively.

- Risk of bias in individual studies

**Figure 1:**
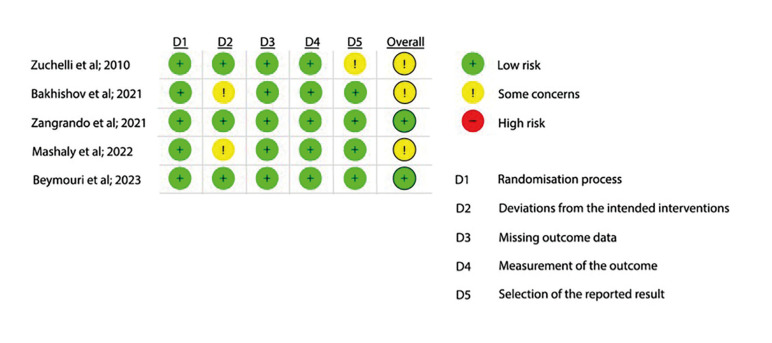
Summary of risk of bias of trials included in SR, according to Cochrane Risk-of-bias tool, RoB2. Plus, sign indicates low risk of bias; minus sign indicates high risk of bias; exclamation mark indicates some concerns for the risk of bias.

An adequate method of sequence generation was reported in all the studies included in this SR. Regarding deviations from intended interventions, 40% of the studies were classified as having a “low” risk of bias (14,37,38). All studies described data outcomes for all participants included in the analysis (8,14,17,37,38). Finally, one study did not report a pre-specified analysis plan before initiation of the study (14). In general, two studies were considered to have a low overall risk of bias (37,38). A summary of bias results is shown in (Fig. 1).

- Clinical results

At 6 months, when DFGG and SCTG were compared, four studies did not find significant differences in terms of a reduction in GR (*p*>0.05) (8,17,37,38). On the other hand, two studies evaluated GR at 12 months (8,14), and one reported a significant difference in favor of the test group (8), while the other study described no significant difference (14). In addition, three and two studies found no significant differences between groups in terms of gain in KTW (8,17,37) at 6 and 12 months (8,17) respectively.

Four studies evaluated CRC at 6 months, (8,17,37,38); one showed a statistically significant difference in favor of DFGG (8) and another showed a significant difference in favor of the control group (*p*=0.001) (38). However, two studies found no significant difference (*p*>0.05) (17,37). On the other hand, two studies evaluated CRC at 12 months of follow-up (8,14), with one study presenting a significant difference in favor of the DFGG group (*p*=0.001) (8), while the other study found no statistically significant differences between the groups (14). In addition, at 6 months postoperatively, four studies evaluated the gain in GT (8,17,37,38). Two did not demonstrate statistically significant differences between the groups (8,37), and two studies showed a significant difference in favor of the DFGG group (*p*<0.05) (17,38). Furthermore, at 12 months two studies evaluated the gain in GT (8,14), with one study reporting a statistically significant difference in favor of the DFGG group (*p*<0.01) (14). In contrast, the other study did not find significant differences between the groups (*p*=0.845) (8). Four of five studies evaluated the gain in CAL; three and two studies did not show a significant difference at 6 (8,17,37) or 12 months (8,14), respectively.

Finally, three studies evaluated the reduction of PD at 6 months (8,17,37). One of the three studies showed that there was a statistically significant difference in favor of the DFGG group (*p*<0.05) (8). However, the other two studies showed no statistically significant differences (17,37). On the other hand, only two studies evaluated the decrease in PD at 12-months, describing no statistically significant differences (8,14).

- Patient-related outcome measurements (PROMS)

The five studies included in this SR evaluated postoperative pain (8,14,17,37,38), which was evaluated using a visual analog scale (VAS) in four studies (8,14,17,37), and in the fifth study it was evaluated using a numeric analog scale (NAS) (38). One of the studies did not demonstrate a statistically significant difference in terms of postoperative pain between DFGG and SCTG at 7 days (14). On the other hand, another study did not show a significant difference between the two groups at 3 days. However, from the third to the seventh day, pain intensity was significantly higher in the DFGG group (*p*<0.05) (38). One study evaluated different follow-up periods of 1, 2, 3, 7, 14, and 28 days, demonstrating no significant differences between the two groups (*p*>0.05) (8). Another study described greater postoperative pain in the DFGG than the SCTG group at 7 days (*p*<0.05) (37). Finally, one study reported a significant difference at 3 days, with the DFGG group presenting greater pain than the SCTG group (*p*<0.05), with no significant difference at 7 days (*p*>0.05) (17).On the other hand, one study evaluated postoperative with a NAS at 3 and 7 days, showing a significant difference between both groups at only 3 days, with the SCTG group presenting less postoperative pain (38).

Only one study evaluated patient satisfaction related to esthetics using a VAS and described an improvement without significant differences between groups at 6 months (*p*>0.05) (37). Finally, one study evaluated dentine hypersensitivity in different follow-up periods after 1, 2, 3, 7, 14, and 28 days using the VAS, and no statistically significant differences were evident between the groups (8).

- Synthesis of meta-analysis results

All the articles reported data on a reduction in GR, and gain in KTW, GT, and CRC, and/or CAL at 6 (8,17,37,38) and 12 months (8,14). Finally, in this SR we also performed meta-analyses of other variables such as PD (Supplement 4). The certainty of evidence of all the outcomes assessed, including the summary of findings Table, are presented in Supplement 5.

Reduction in GR

**Figure 2:**
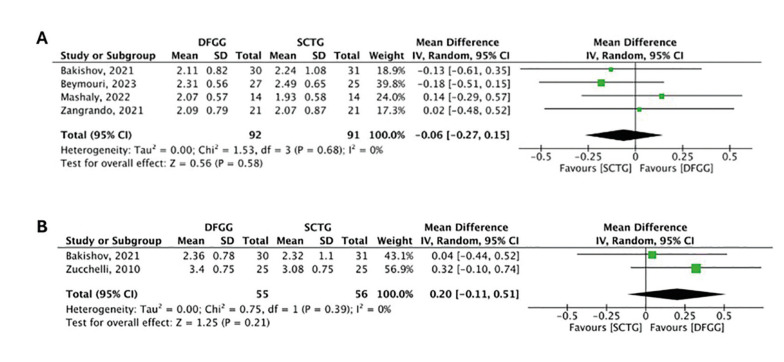
Comparison of reduction in gingival recession between subepithelial connective tissue graft (SCTG) and de-epithelialized free gingival graft (DFGG) at 6 months (A) and 12 months (B).

Four studies evaluated the reduction in GR at 6 months of follow-up. The meta-analysis did not show statistically significant differences between groups (MD: -0.06; 95% confidence interval [CI]: -0.27 - 0.15), presenting low heterogeneity (I2= 0%, *p*= 0.68) (Fig. 2) (certainty of evidence: very low). Two studies evaluated the reduction in GR at 12 months of follow-up. The meta-analysis did not show statistically significant differences between groups (MD: 0.20; 95% CI: -0.11 - 0.51), presenting low heterogeneity (I2= 0%, *p*= 0.39) (Fig. 2) (certainty of evidence: very low).

Gain in KTW

**Figure 3:**
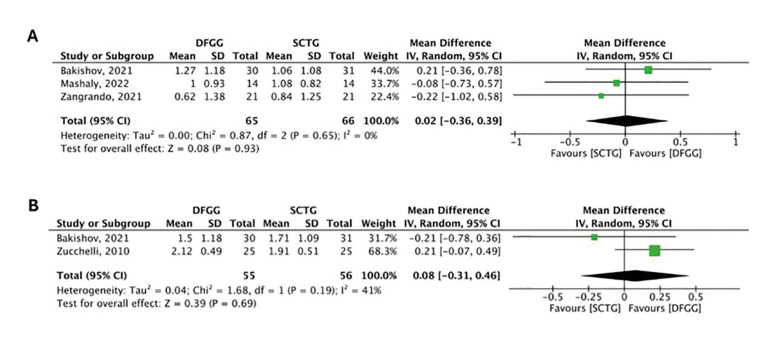
Comparison of gain in keratinized tissue width between subepithelial connective tissue graft (SCTG) and de-epithelialized free gingival graft (DFGG) at 6 months (A) and 12 months (B).

Fig. 3 shows the forest plot that included three studies which evaluated the gain in KTW between SCTG and DFGG at 6 months of follow-up. The meta-analysis did not demonstrate statistically significant differences between the two groups (MD: 0.02; 95%CI: -0.36 - 0.39), showing low heterogeneity (I2 = 0%, *p*= 0.65) (certainty of evidence: very low). In Fig. 3, at 12 months of follow-up, two studies did not demonstrate statistically significant differences in both groups (MD: 0.08; 95%CI: -0.31 - 0.46), showing moderate heterogeneity (I2 = 41%, *p*= 0.19) (certainty of evidence: very low).

Gain in GT

**Figure 4:**
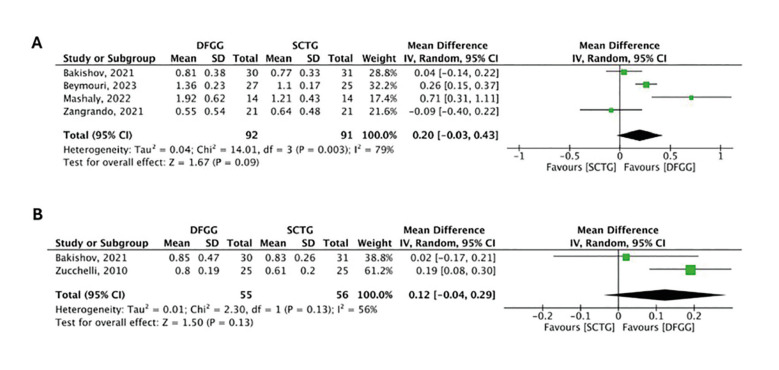
Comparison of gain in gingival thickness between subepithelial connective tissue graft (SCTG) and de-epithelialized free gingival graft (DFGG) at 6 months (A) and 12 months (B).

Four studies evaluated the gain in GT between SCTG and DFGG at 6 months of follow-up. The meta-analysis did not demonstrate statistically significant differences between the two groups (MD: 0.20; 95%CI: -0.03 - 0.43), showing high heterogeneity (I2 = 79%, *p*= 0.003) (Fig. 4) (certainty of evidence: very low). At 12 months of follow-up, two studies did not demonstrate statistically significant differences between the two groups (MD: 0.12; 95%CI: -0.04 - 0.29), showing moderate heterogeneity (I2 = 56%, *p*= 0.13) (Fig. 4) (certainty of evidence: very low).

CRC

For this outcome, the meta-analysis did not demonstrate statistically significant differences between both groups 12% (relative risk [RR]: 1.02; 95% CI: 0.78 - 1.32), showing low heterogeneity (I2 = 36%, *p*=0.19) at 6 months (Supplement 6) (certainty of evidence: very low), and 20% (RR: 1.20; 95% CI: 0.96 - 1.51), showing low heterogeneity (I2 = 0%, *p* = 0.76) at 12 months (Supplement 6) (certainty of evidence: very low).

Gain in CAL

Three studies evaluated the gain in CAL at 6 months of follow-up. The meta-analysis did not show statistically significant differences between groups (MD: -0.04; 95% CI: -0.37 - 0.28), presenting low heterogeneity (I2= 0%, *p*= 0.54) (certainty of evidence: very low) (Supplement 7). Two studies evaluated the gain in CAL at 12 months of follow-up, with no statistically significant differences being shown between groups (MD: 0.12; 95% CI: -0.21 - 0.45) and presenting low heterogeneity (I2= 0%, *p*= 0.38) (certainty of evidence: very low) (Supplement 7).

## Discussion

This SR aimed to compare the clinical effects of SCTG and DFGG in the treatment of Miller Class I, II and Cairo Type I GR. The efficacy of SCTG in the treatment of GR has been widely demonstrated and is considered the gold standard for the treatment of single GR (39). In contrast, DFGG has shown additional benefits, such as a greater increase in the thickness of the grafted area (17,38).

The main objective of this SR was to identify the difference in terms of the reduction in GR achieved comparing the two techniques. Our findings indicate that both SCTG and DFGG produce a significant reduction in terms of GR depth and gain in KTW, GT, CRC, and CAL, with no differences between groups at 6 and 12 months. The results of our review do not agree with the SR by Tavelli *et al*. in 2019 (15), who found slightly superior results in terms of GR reduction, KTW gain, PD reduction and CAL gain using CAF + DFGG compared to CAF + SCTG. However, they concluded that the evidence available in the literature was limited. For this reason, our SR aimed to update this information, also providing direct comparisons of the available literature, decreasing the risk of indirectness.

In terms of CRC, this review found no significant differences between the two groups. Beymouri *et al*. (38) demonstrated statistically better results for the SCTG+CAF group. On the other hand, Mashaly *et al*. (17) demonstrated the efficacy of SCTG and DFGG with CAF reporting that both approaches achieved complete root coverage of 92.9%, Zucchelli *et al*. (14) found high CRC percentages with no significant difference between the use of SCTG and DFGG. Only Bakhishov *et al*. (8) found that DFGG combined with TUN resulted in a greater reduction in GR and CRC compared to SCTG for multiple GRs, although the differences were not statistically significant. These results are in agreement with the SR of Tavelli *et al*.(15), who reported superior results using DFGG in terms of a reduction in GR and other clinical parameters when used with CAF. However, a SR by Konflanz *et al*. (40) indicated that if CRC is the primary clinical objective, extraoral de-epithelialization may be preferable to intraoral de-epithelialization techniques. The superiority of DFGG was also associated with the quality of the connective tissue in terms of stability and lower shrinkage resulting from DFGG compared to that obtained with the Trap-door technique (14).

Other variables such as GR depth, KTW and GT may influence treatment outcomes. Deeper recessions and thicker GT have been found to be associated with better root coverage outcomes (8). Although three studies included in our review showed statistically significant differences regarding GT 6 months after DFGG + CAF, the meta-analysis failed to demonstrate the same trend of results (14,17,38).

Postoperative morbidity can include discomfort, bleeding, swelling and even pain. In this SR both techniques showed similar results in terms of postoperative morbidity. However, some studies suggest that DFGG may be associated with greater postoperative discomfort compared to SCTG. The removal of a SCTG is aimed at primary closure of the palatal flap and is classified according to the number of vertical incisions. On the other hand, DFGG involves the extraction of a graft that leaves an open wound that will heal by secondary closure (15,17). The studies by Mashaly *et al*. (17) and Beymouri *et al*. (38) showed that patients treated with DFGG reported higher stress scores and inability to chew than those treated with SCTG, although overall satisfaction was comparable between the two groups (14).

In addition, it has been reported that the level of pain and analgesic consumption was higher with the DFGG+CAF compared to SCTG+CAF approach (38). However, analgesic consumption was found to increase when using wide grafts and when complications, such as tissue necrosis, occur in the donor site (14).

- Limitations and future direction

The present SR was intended to update the search for previous publications. However, the main limitation of the present SR is the small number of RCTs obtained, since many were excluded for not fulfilling the inclusion criteria. The results of the present SR must be interpreted with caution. First of all, it should be kept in mind that in this meta-analysis, the outcomes of root coverage surgery were performed in different defects (i.e., single-, and multiple gingival recessions) as well as in different types of randomized clinical studies such as parallel and split-mouth.

Further studies should focus on long-term comparative studies with standardized outcome measures to better elucidate the relative benefits and drawbacks of DFGG and SCTG. In addition, the biological mechanisms underlying the healing processes of both approaches could be explored to optimize these treatments for GR. It would be interesting to include histological evaluation in future studies to reveal differences in cellularity and revascularization between DFGG and SCTG, which could influence the healing process and final results (8).

Some observations on the applicability of the results obtained can be formulated. The use of DFGG does not develop any additional benefit in comparison with SCTG. More well-designed randomized clinical trials are needed to confirm these results.

The present study endeavored to summarize the best evidence available but not always the least biased. The limitations of evidence were comprehensively summarized in a transparent manner using the GRADE approach, according to the most recent recommendations stated in the Cochrane and non-Cochrane systematic reviews [50]. Further studies are warranted to increase the body of evidence accumulated, considering the above-mentioned limitations.

## Conclusions

Within the limitations of this study, the present SR found that both DFGG and SCTG are effective in the treatment of GR at 6 and 12 months.

## Figures and Tables

**Table 1:** Characteristics and main results of the studies included.

Author, Year (Country)	Study design	Defect	Subjects	M/FAge	Interventions (n of teeth)	Clinical parameters	Patient reported outcomes	Follow-up (Months)	Main findings
Zuchelli et al. 2010 (Italy)	RCT Parallel	GR≥2mm Miller class I or II Maxilla Mandible	50 patients with 50 recession defects	22/28 21 - 50	Control group CAF + SCTG (n=25) Test group CAF + DFGG (n=25)	GR, PD, CAL, KTW, GT, CRC	Postoperative pain (VAS)	12	There were no significant differences regarding GR reduction, CRC, PD reduction, CAL, KTW gain and postoperative pain between groups at 12 months (p>0.05). However, the DFGG group showed greater gain in GT than the SCTG group at 12 months (p<0.05).
Zangrando et al. 2020 (Brazil)	RCTSplit-mouth	GR depth ≥2 mmMiller class I or II (RT1) MaxillaMandible	21 patients with 84 recession defects	8/13 25 - 54	Control group CAF + SCTG (n=42) Test group CAF + DFGG (n=42)	GR, PD, CAL, KTW, GT, CRC	Postoperative pain, aesthetic satisfaction (VAS)	6	Both groups showed no significant differences in GR reduction, CRC, KTW, CAL and GT gain at 6 months of follow-up (p>0.05). Besides, both techniques improved esthetics evaluated by patients, without significant differences. On the other hand, SCTG group presented inferior pain/discomfort compared with DFGG group (p<0.05).
Bakhishov et al, 2021 (Turkey)	RCT Parallel	GR depth ≥2 mm Miller class I or II (RT1) MaxillaMandible	27 patients with 61 recession defects	15/12 18 - 60	Control group TUN + SCTG (n = 31) Test group TUN + DFGG (n = 30)	GR, PD, CAL, KTW, GT, CRC	Postoperative pain	6 and 12	The DFGG group showed a significant difference regarding GR reduction and CRC than the SCTG group at 12 months (p=0.001). However, there were no significant differences in PD reduction, CAL, KTW, GT gain and postoperative pain.
Mashaly et al, 2022 (Egypt)	RCT	GR≥2mm Miller class I or II Maxilla	28 patients with 28 recession defects	11/17 22 - 37	Control group CAF + SCTG (n=14) Test group CAF + DFGG (n=14)	GR, PD, CAL, KTW, GT,	Postoperative pain	3 and 6	There were no significant differences regarding GR reduction, PD reduction, CAL and KTW gain between groups at 6 months (p>0.05). However, the DFGG group showed greater gain in GT and postoperative pain than the SCTG group (p<0.05).
Beymouri et al, 2023 (Iran)	RCT Split-mouth	GR ≥2 mm Miller class I or II Maxilla Mandible	12 patients with 52 recession defects	4/8 19 - 61	Control group CAF + SCTG (n=25) Test group CAF + DGG (n=27)	GR, GT, CRC	Postoperative pain (NAS)	6	At 6 months of follow-up, there were no significant differences regarding GR reduction. On the other hand, the DFGG group showed greater GT gain compared to the SCTG group (p=0.001). However, regarding CRC, there was a significant difference in favor of the SCTG group.

CAF: coronally advanced flap; CAL: clinical attachment level; CRC: complete root coverage; CTG: connective tissue graft; F: female; GR: gingival recession; GT: gingival thickness; KTW: keratinized tissue width; M: male; NR: not reported; RCT: randomized clinical trial; RES: Root coverage aesthetic score; TUN: tunnel technique; VAS: visual analog scale; NAS: numeric analog scale.
